# Piperlongumine alleviates corneal allograft rejection *via* suppressing angiogenesis and inflammation

**DOI:** 10.3389/fimmu.2022.1090877

**Published:** 2022-12-16

**Authors:** Xiangyu Fan, Jini Qiu, Tianjie Yuan, Jing Zhang, Jianjiang Xu

**Affiliations:** ^1^ Eye Institute and Department of Ophthalmology, Eye & ENT Hospital, Fudan University, Shanghai, China; ^2^ Key Laboratory of Myopia, Chinese Academy of Medical Sciences, Shanghai, China; ^3^ Shanghai Key Laboratory of Visual Impairment and Restoration, Fudan University, Shanghai, China; ^4^ National Health Commission (NHC), Key Laboratory of Myopia (Fudan University), Chinese Academy of Medical Sciences, Shanghai, China

**Keywords:** piperlongumine, corneal allograft rejection, angiogenesis, inflammation, hypoxia

## Abstract

**Background:**

Neovascularization and inflammatory response are two essential features of corneal allograft rejection. Here, we investigated the impact of Piperlongumine (PL) on alleviating corneal allograft rejection, primarily focusing on pathological angiogenesis and inflammation.

**Methods:**

A murine corneal allograft transplantation model was utilized to investigate the role of PL in preventing corneal allograft rejection. PL (10 mg/kg) or vehicle was intraperitoneally injected daily into BALB/c recipients from day -3 to day 14. The clinical signs of the corneal grafts were monitored for 30 days. Corneal neovascularization and inflammatory cell infiltration were detected by immunofluorescence staining and immunohistochemistry. The proportion of CD4^+^ T cells and macrophages in the draining lymph nodes (DLNs) was examined by flow cytometry. *In vitro*, HUVECs were cultured under hypoxia or incubated with TNF-α to mimic the hypoxic and inflammatory microenvironment favoring neovascularization in corneal allograft rejection. Multiple angiogenic processes including proliferation, migration, invasion and tube formation of HUVECs in hypoxia with or without PL treatment were routinely evaluated. The influence of PL treatment on TNF-α-induced pro-inflammation in HUVECs was investigated by real-time PCR and ELISA.

**Results:**

*In vivo*, PL treatment effectively attenuated corneal allograft rejection, paralleled by coincident suppression of neovascularization and alleviation of inflammatory response. *In vitro*, PL distinctively inhibited hypoxia-induced angiogenic processes in HUVECs. Two key players in hypoxia-induced angiogenesis, HIF-1α and VEGF-A were significantly suppressed by PL treatment. Also, TNF-α-induced pro-inflammation in HUVECs was hampered by PL treatment, along with a pronounced reduction in ICAM-1, VCAM-1, CCL2, and CXCL5 expression.

**Conclusions:**

The current study demonstrated that PL could exhibit both anti-angiogenic and anti-inflammatory effects in preventing corneal allograft rejection, highlighting the potential therapeutic applications of PL in clinical strategy.

## 1 Introduction

Corneal transplantation is a crucial means of visual rehabilitation, while the loss of corneal immune privilege accompanied by allograft rejection remains to be the major barrier to the success of transplantation (1). The “immune privilege” can be disrupted by corneal neovascularization (CoNV), including both hemangiogenesis and lymphangiogenesis, which is predominantly driven by inflammation and hypoxia (2). One of the most important functions of CoNV is believed to be structural components of the corneal immune reflex arc (3, 4). Specifically, serving as an afferent arm, lymph vessels assist the transport of antigen-presenting cells (APCs) and antigenic materials to the regional lymph nodes and promote antigen presentation at this site. Subsequently, activated T cells and inflammatory mediators are recruited and attacked the graft through efferent blood vessels, and consequently mediate an inflammatory response that eventually cause corneal allograft rejection (5, 6). CoNV and inflammation are two interplaying key factors determining the progression of corneal allograft rejection. Inflammation is widely recognized as an inducer for CoNV, in turn, CoNV can act as an amplifier of inflammation. Illustratively, inflammatory stimuli can not only drive the angiogenic response, but also induce vascular endothelial cells to express adhesion molecules and secret chemokines, resulting in recruitment of leukocytes and exacerbation of the inflammation (7–9).

Piperlongumine (PL) is a biologically active alkaloid isolated from the long pepper (Piper longum) that has various pharmacological properties, including anti-tumoral, anti-depressant, anti-diabetic, anti-atherosclerotic and neuroprotective properties (10, 11). The inhibition of PL on tumor angiogenesis has been confirmed by an alginate-encapsulated tumor cell assay in a previous study^11^. Its effectiveness in inhibiting inflammation has been proven by research focusing on immune-mediated inflammatory diseases, like asthma or rheumatoid arthritis (12, 13). However, the application of PL in the transplantation field, especially its role in corneal allograft rejection, is entirely unknown. Whether PL could modulate CoNV and inflammation during the process of corneal allograft rejection remains to be elucidated.

The aim of the current study was to investigate the potential of PL treatment for corneal allograft rejection. The therapeutic effects of PL were first evaluated in the murine corneal allograft transplantation model in terms of suppressing corneal neovascularization and inhibiting the inflammatory response. Moreover, human umbilical vein endothelial cells (HUVECs) were cultured under hypoxic and TNF-α stimulation conditions, to mimic the hypoxic and inflammatory microenvironment in corneal allograft rejection. The impact of PL treatment on inhibiting angiogenesis and pro-inflammation mediated by vascular endothelial cells in corneal allograft rejection and the underlying mechanisms were further investigated.

## 2 Materials and methods

### 2.1 Allogeneic murine corneal transplantation model

All animal experimental procedures were approved by the Animal Care and Use Committee of Eye & ENT Hospital (Shanghai, China), were conducted in accordance with the ARVO Statement for the Use of Animals in Ophthalmic and Vision Research. Allogeneic and syngeneic murine corneal transplantation model were constructed as previously described by our laboratory (14). Mice receiving allogeneic transplantation were randomized and divided into vehicle-treated mice and PL-treated mice. PL-treated mice received the PL solutions (10 mg/kg/d), and vehicle-treated mice received equal amounts of the vehicle (PBS containing DMSO with 0.1% final DMSO concentration). From three days before corneal transplantation to the 14th day after transplantation, the two groups of mice were intraperitoneally injected with PL or vehicle solutions every day. Corneal grafts were observed under a slit lamp microscope for 30 days. The degree of corneal opacity and neovascularization were evaluated in compliance with a standardized scoring system to assess opacity (range, 0-5+) and neovascularization score (range, 0-8+) (15). The opacity scoring system (range, 0-5+) was illustrated as follows: 0  =  clear graft; 1+  =  minimal non-stromal opacity with clear observation of the pupil margin and iris vessels; 2+  =  minimal stromal opacity with observation of the pupil margin and iris vessels; 3+  =  moderate stromal opacity with observation of the pupil margin only; 4+  =  intense stromal opacity with observation of a portion of the pupil margin; 5+  =  maximum stromal opacity  with total obscuration of the anterior chamber. Grafts with corneal opacity score of more than 2 were defined as rejection. Graft survival rates were plotted as Kaplan–Meier curves.

### 2.2 Immunofluorescence staining and morphometric analysis of hemangiogenesis and lymphangiogenesis in flat-mounted corneas

The eyeballs from the two groups were harvested on the 14th day after surgery. Corneas were dissected and flattened with four radial incisions. After being fixed with 4% paraformaldehyde for 30 min, the samples were permeabilized with 0.3% Triton X-100 at room temperature for 30 min and blocked with 3% donkey serum for 1 h. Thereafter the corneas were stained overnight at 4°C with goat anti-mouse CD31 antibody (1;100, AF3628, R&D Systems) and rabbit anti-mouse LYVE-1 antibody (1;100, ab33682, Abcam). After three rinses in PBS, anti-CD31 antibody and anti-LYVE-1 antibody were detected with anti-goat IgG Alexa Fluor 488 (A-11055, Invitrogen Antibodies) and FITC-conjugated anti-rabbit IgG (1;1000, SA00003-2, Proteintech), respectively. Nuclei were visualized using DAPI staining. Images of the flat-mount stained corneas were captured with a confocal microscope (Leica Microsystems, Germany) and morphometric analysis was performed using the ImageJ software. The area of corneal neovascularization (CD31 positive for hemangiogenesis and LYVE-1 positive for lymphangiogenesis) was quantified and normalized to the total corneal area.

### 2.3 Immunohistochemistry

On day 14 after surgery, all samples were immediately fixed with 4% PFA, embedded in paraffin, and sectioned serially. Paraffin-embedded sections were deparaffinized and rehydrated, and then subjected to heat-mediated antigen retrieval. Subsequently, the sections were incubated with rabbit anti-mouse CD4 antibody (1:100, ab183685, Abcam), rabbit anti-mouse CD8 antibody (1:100, ab4055, Abcam), and rabbit anti-mouse F4/80 antibody (1:100, ab111101, Abcam) at 4°C overnight. Upon incubation with HRP-conjugated secondary antibodies, proteins were visualized using diaminobenzidine chromogen (DAB).

### 2.4 Western blot analysis

Protein lysates from the corneal allograft tissues and cell lysates were separated by sodium dodecyl sulfate-polyacrylamide gel electrophoresis (SDS-PAGE) and transferred to PVDF membranes. Following blockage of nonspecific binding sites with 5% skimmed milk, the membranes were incubated overnight at 4°C with primary antibody anti-HIF-1α (1:500, #36169, Cell Signaling Technology). HRP-conjugated goat anti-rabbit IgG (1:1000, #7074, Cell Signaling Technology) was used as a secondary antibody in standard ECL analysis (Thermo Fisher Scientific, USA). β-Actin served as a loading control to ensure an equal amount of protein loaded.

### 2.5 Quantitative real-time PCR

Total RNA from murine corneas was isolated using the RNA simple Total RNA Kit (TIANGEN, China), and first-stranded cDNA was synthesized by the FastQuant RT Kit (TIANGEN, China) according to the manufacturer’s protocol. Quantitative real-time PCR (qRT-PCR) was performed using the QuantiNova SYBR Green PCR Kit (Qiagen, Germany), and the expressions of genes were normalized to the GAPDH expression. The primer sequences used in the experiment were summarized in **Table 1**.

**Table 1:** Primers used for real time-PCR.

Gene name	Species	Orientation	Primer sequence (5’ - 3’)
GAPDH	Mouse	forward	AATGGATTTGGACGCATTGGT
		reverse	TTTGCACTGGTACGTGTTGAT
IL-1β	Mouse	forward	TGAAGTTGACGGACCCCAAA
		reverse	TGATGTGCTGCTGGGAGATT
IL-1α	Mouse	forward	TGCCATTGACCATCTCTCTCTG
		reverse	TGGCAACTCCTTCAGCAACACG
TNF-α	Mouse	forward	AATGGCCTCCCTCTCATCAGT
		reverse	GCTACAGGCTTGTCACTCGAATT
IL-17	Mouse	forward	GCTCCAGAAGGCCCTCAG ACT
		reverse	CCAGCTTTCCCTCCGCATTGA
IFN-γ	Mouse	forward	CGGCACAGTCATTGAAAGCCTA
		reverse	AGGGCTGCTTTAACTCTGGT
VEGF-A	Mouse	forward	CAGCTATTGCCGTCCGATTGAGA
		reverse	TGCTGGCTTTGGTGAGGTTTGAT
VEGF-C	Mouse	forward	AGCTGAGGTTTTTCTCTTGTGATTTAA
		reverse	TGATCACAGTGAGCTTTACCAATTG
MMP-2	Mouse	forward	ATGCCATCCCTGATAACCTG
		reverse	CCCAGCCAGTCTGATTTGAT
MMP-9	Mouse	forward	TCTTCTGGCGTGTGAGTTTCC
		reverse	CGGTTGAAGCAAAGAAGGAGC
GAPDH	Human	forward	GCACCGTCAAGGCTGAGAAC
		reverse	TGGTGGTGAAGACGCCAGT
VEGF-A	Human	forward reverse	AGGGCAGAATCATCACGAAGT AGGGTCTCGATTGGATGGCA
VEGF-C	Human	forward	GGCTGGCAACATAACAGAGAA
		reverse	CCCCACATCTATACACACCTCC
MMP-2	Human	forward	TGGCAAGTACGGCTTCTGTC
		reverse	TTCTTGTCGCGGTCGTAGTC
MMP-9	Human	forward	TGCGCTACCACCTCGAACTT
		reverse	GATGCCATTGACGTCGTCCT
Hif-1α	Human	forward	GAACGTCGAAAAGAAAAGTCTCG
		reverse	CCTTATCAAGATGCGAACTCACA
ICAM-1	Human	forward	GAACCAGAGCCAGGAGACAC
		reverse	TCCCTTTTTGGGCCTGTTGT
VCAM-1	Human	forward	CAAATCCTTGATACTGCTCATC
		reverse	TTGACTTCTTGCTCACAGC
CCL2	Human	forward	CAGCGACATGCAATCAATGC
		reverse	GTGGTCCATGGAATCCTGAA
CXCL5	Human	forward	AGCTGCGTTGCGTTTGTTTAC
		reverse	TGGCGAACACTTGCAGATTAC

### 2.6 Flow cytometry analysis

On day 14 post-surgery, single-cell suspension was prepared from draining submandibular and cervical lymph nodes. The expression of CD4^+^ T cells and macrophages in the peripheral lymph nodes were analyzed using flow cytometry. Upon resuspension in binding buffer at a density of 2 × 10^7^ cells/ml, the cells were then stained with APC-CY7 conjugated anti-mouse CD45 (#561037, BD Biosciences), AF700 conjugated anti-mouse CD3 (#557984, BD Biosciences) and FITC conjugated anti-mouse CD4 (#561831, BD Biosciences) to label the surface markers of CD4^+^ T cells. The surface markers of macrophages were detected by APC-conjugated anti-mouse CD11B (#561039, BD Biosciences) and PE-conjugated anti-mouse F4/80 (#565410, BD Biosciences).

### 2.7 Cell culture

Human Umbilical Vein Endothelial Cells (HUVECs) were cultured in a humidified atmosphere containing 5% CO2 at 37°C in Endothelial Growth Medium-2 (EGM2, Lonza, Switzerland). For hypoxia induction, cells were incubated in a hypoxia chamber with a mixture of 1% O2, 5% CO2, and 94% N2. For TNF-α stimulation, 10 ng/ml of recombinant human TNF-α (BD Biosciences, USA) was added into the HUVECs medium. Piperlongumine (Sigma-Aldrich, USA) was dissolved in dimethylsulfoxide (DMSO, Sigma-Aldrich, USA) before usage. The culture medium was replaced every 1-2 days, and cells at 85-90% confluence were passaged at a ratio of 1:2 confluence.

### 2.8 CCK-8 analysis

Cell Counting Kit-8 (CCK-8; Dojindo, Japan) was used to detect cell viability. Briefly, HUVECs and HCECs were seeded into 96-well plates (5.0×10^3^ cells/well) in quintuplicate, and allowed to adhere for 4 h. After the required treatments, HUVECs and HCECs were added with CCK-8 reagent (10μl/well) and incubated at 37°C for an additional 2 h. The optical density (OD) at 450 nm was measured using an automatic microplate reader (BioTek, USA). Three identical replicates were performed.

### 2.9 Wound-healing assay

A two-well silicone culture insert (Ibidi, Germany) with a defined cell-free gap, suitable for migration assays, was used as a migration barrier. The two-well silicone culture insert was then placed in the middle of the plate. Subsequently, cells at a density of 5 × 10^5^/ml were seeded onto the culture plate 70μL per well and allowed to proliferate to 100% confluence under standard laboratory conditions. After attachment, the silicone insert was removed, leaving a 500μm cell-free gap. Culture medium with different treatments was added respectively. Finally, an inverted microscope (Zeiss, Germany) was equipped to capture the migration distance of HUVECs to the cell-free zone during 12h. ImageJ software was used to measure the areas of the wound.

### 2.10 Transwell invasion assay

Cell migration assay was performed in a 24-well Transwell chamber (8.0 μm pore size, Corning, USA) pre-coated with matrigel. HUVECs (1×10^6^/ml) suspended in 200 μl DMEM (Hyclone, USA) were added to the upper compartment. In addition, 600 µl EGM-2 with the required treatment was added to the bottom chamber. Following incubation at 37°C for 24 h, non-migrating cells were removed from the upper surface by gentle scrubbing. Migrating cells attached to the lower membrane were fixed with 4% paraformaldehyde and stained with 0.1% crystal violet (Aladdin, China). Data are expressed as an average number of cells per field that migrated through pores.

### 2.11 Tube formation assay

Tube formation assay was applied to detect the ability of endothelial cells to generate vessels. Briefly, a 96-well plate coated with 50 µl Matrigel (BD Biosciences, USA) was incubated in a cell incubator for 1 hour. Then, HUVECs were seeded into the Matrigel-coated wells in triplicate, and cultured at 37°C for 12 hours. Next, an inverted phase contrast microscope (Zeiss, Germany) was used to observe the formation of tubes. The number of tubes and their length were quantified using Image J analyzer.

### 2.12 ELISA

The expressions of pro-inflammatory chemokines (CCL2 and CXCL5) in the supernatant containing the HUVECs lysates were measured using ELISA kits from R&D Systems (DCP00 and DY254) following the manufacturer’s instruction. The levels of adhesion molecules (ICAM-1 and VCAM-1) were evaluated with ELISA kits from Abcam (ab174445 and ab100661). Protein concentrations were calculated from the standard curves and were expressed as pg/ml.

### 2.13 Statistical analysis

Data are presented as Mean ± SEM of three independent experiments. Statistical differences between control and experimental groups were determined by unpaired two-tailed Student’s t-test. Comparisons among multiple groups were analyzed by one-way ANOVA with Bonferroni corrections. Overall survival analyses of corneal allografts were performed using the Kaplan-Meier method (Log-rank test). The statistical analyses were performed using the SPSS 20.0 software, and the P-value < 0.05 was considered statistically significant.

## 3 Results

### 3.1 PL improved corneal graft survival in murine model

**Figure 1:**
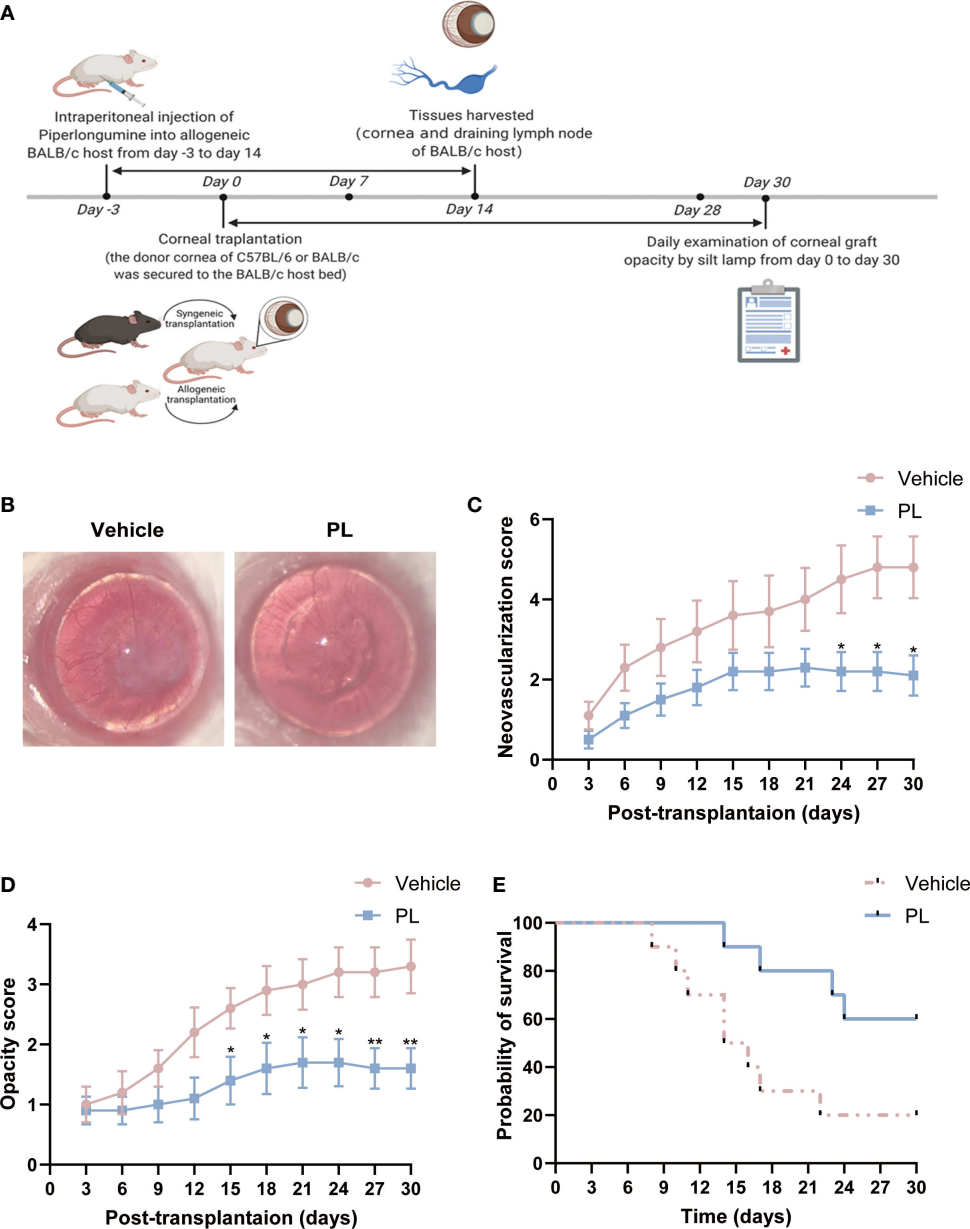
PL suppressed corneal allograft rejection in a murine model. **(A)** A schematic diagram depicts the experimental design for the evaluation of the therapeutic effect of PL in the murine corneal allograft transplantation model. Allogeneic BALB/C host in PL-treated group were injected intraperitoneally with PL solutions (10 mg/kg/d) from day 3 before surgery till day 14 after surgery, whereas mice in vehicle-treated group were treated with an equal volume of sterile saline. Mice in two groups were observed daily for 30 days after surgery. **(B)** Representative slit-lamp images of corneal grafts from two groups on postoperative day 30. **(C)** Neovascularization score of corneal grafts from two groups were analyzed by serial observation (n = 10/group). **(D)** Opacity score of corneal grafts from two groups were analyzed by serial observation (n = 10/group). **(E)** Kaplan-Meier survival curve of corneal grafts from two groups were analyzed by serial observation (n = 10/group). The data are presented as mean ± SEM. *P < 0.05, **P < 0.01. Panel **(A)** from BioRender.

A murine model of allogeneic corneal transplantation was constructed by transplanting corneal grafts from C57BL/6 mice to BALB/c mice. PL (10 mg/kg) or an equal volume of vehicle was intraperitoneally injected daily into allogenic BALB/c recipient from day -3 to day 14 (**Figure 1A**). To determine whether PL could prolong allograft survival, the corneal opacity and angiogenesis were compared between vehicle-treated mice and PL-treated mice. Vehicle-treated mice exhibited conspicuous corneal edema and stromal opacity, with neovessels growing from the limbus towards the graft at postoperative day 30. In contrast, the corneal grafts of PL-treated mice mostly maintained transparent or mildly turbid (**Figure 1B**). Compared to the vehicle-treated mice, PL treatment was capable of suppressing the degree of corneal neovascularization. Such suppressive effect became significant since day 24 post-transplantation till the end point of the observation (**Figure 1C**, P<0.05). Likewise, PL-treated mice had lower graft opacity scores than vehicle-treated mice; as presented in **Figure 1D**, a more significant reduction in the graft opacity score was observed with the prolongation of PL treatment time. Additionally, Kaplan-Meier survival curves showed that PL treatment could achieve almost 60% 30-day graft survival (mean survival time [MST] of 25.80 ± 1.93 days), which was significantly higher than 30-day graft survival rate of nearly 20% (MST of 17.20 ± 2.47 days) in vehicle-treated mice (**Figure 1E**).

### 3.2 PL suppressed CoNV in murine corneal allograft transplantation model

**Figure 2:**
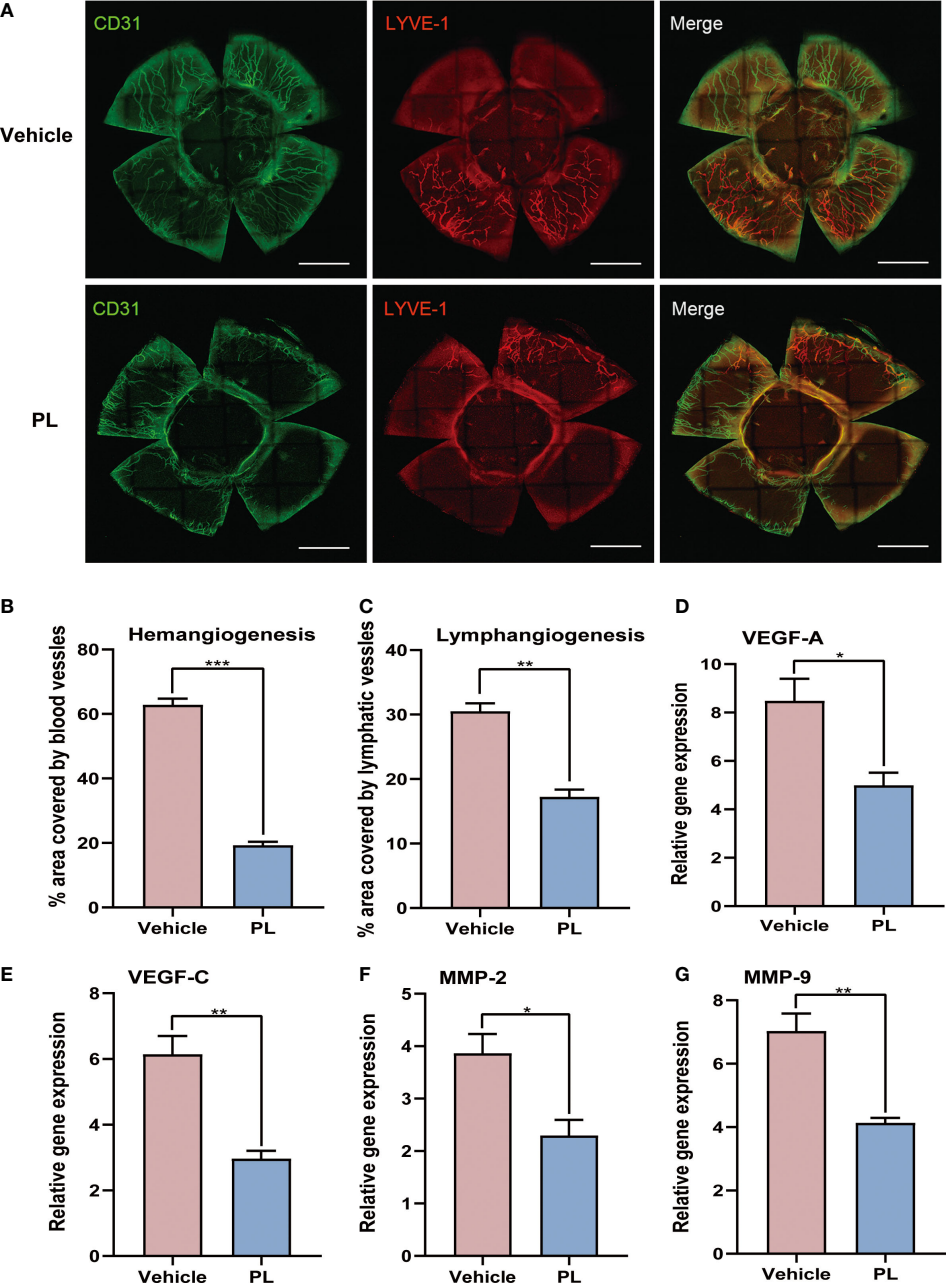
PL suppressed coNV in murine corneal allograft transplantation model. **(A)** Representative immunofluorescence of corneal flat mounts showing ingrowth of CD31^+^ blood vessels and LYVE-1^+^ lymphatic vessels from vehicle-treated group and PL-treated group on postoperative day 14. **(B, C)** Statistical quantification of the area covered by CD31^+^ blood vessels **(B)** and LYVE-1^+^ lymphatic vessels **(C)** from two groups on postoperative day 14 (n = 4/group). scale bar 1 mm. **(D-G)** The mRNA levels of several pro-angiogenic and pro-lymphangiogenic factors were determined by qRT-PCR (n = 6/group). Data were normalized to the expression of these genes in untreated BALB/c mice. *P < 0.05, **P < 0.01, ***P < 0.001.

To determine whether PL treatment could ameliorate corneal neovascularization, corneas from vehicle-treated and PL-treated mice were dissected, and the area invaded by blood vessels (BVs) and lymphatic vessels (LVs) was quantified by fluorescent staining. As shown in **Figure 2A**, the radial growth of blood vessels that invaded the corneal grafts can be found in all four quadrants of the corneas of vehicle-treated mice. In contrast, the corneas of PL-treated mice presented significant amelioration on corneal neovascularization, shown as a substantially decreased percentage of area covered by new BVs and LVs, which only existed in host corneal beds (**Figures 2A–C**, *P* < 0.001 for BVs, *P* < 0.01 for LVs). Consistently, PL treatment markedly decreased the mRNA levels of pro-angiogenic factors, including VEGF-A (**Figure 2D**, *P* < 0.05), VEGF-C (**Figure 2E**, *P* < 0.01), MMP-2 (**Figure 2F**, *P* < 0.05) and MMP-9 (**Figure 2G**, *P* < 0.01), compared with vehicle-treated mice. Among these, VEGF-C could regulate both angiogenesis and lymphangiogenesis.

### 3.3 PL alleviated inflammatory response in murine corneal allograft transplantation model

**Figure 3:**
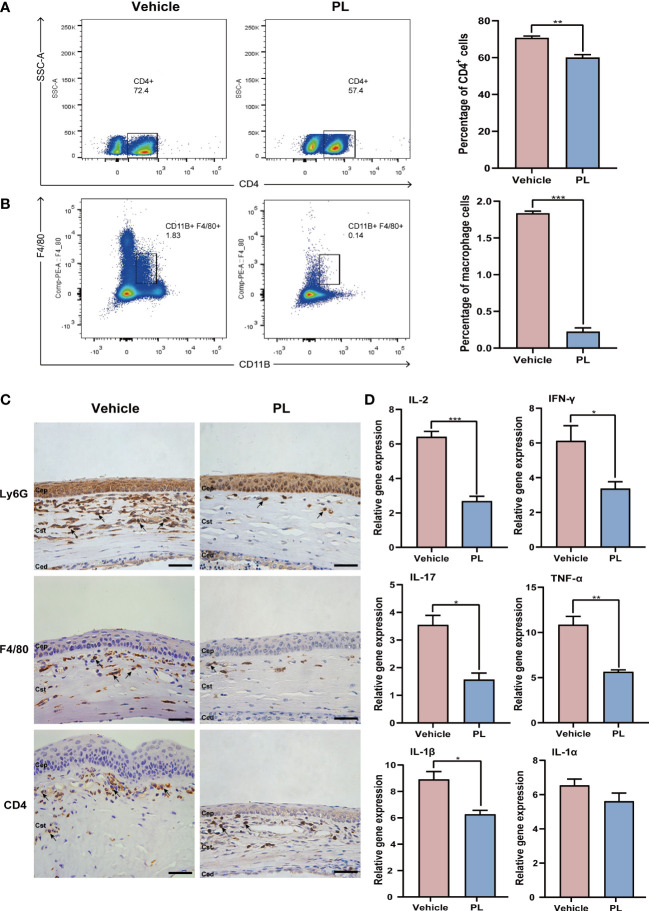
PL alleviated inflammatory response in murine corneal allograft transplantation model. **(A)** Representative flow cytometry plots and statistical analysis of CD4^+^ T cell frequencies from vehicle-treated group and PL-treated group on postoperative day 14. (n = 10/group). **(B)** Representative flow cytometry plots and statistical analysis of CD11B^+^F4/80^+^ macrophage frequencies from two groups on postoperative day 14 (n = 10/group). **(C)** Representative immunohistochemistry of Ly6G^+^ neutrophils, CD4^+^ T cells and CD11B^+^F4/80^+^ macrophages in the cornea from two groups on postoperative day 14 (n = 4/group). scale bar 100 µm. **(D)** The mRNA levels of inflammatory cytokines were detected by qRT-PCR (n = 6/group). *P < 0.05, **P < 0.01, ***P < 0.001.

On day 14 after transplantation, the DLNs of recipients were collected, CD4^+^ T cells and macrophages were sorted by flow cytometry. The result showed that PL treatment significantly decreased the percentage of CD4^+^ T cells (**Figure 3A**, 60.10 ± 1.56% vs. 70.70 ± 1.01%, *P* < 0.01) and macrophages (**Figure 3B**, 0.22 ± 0.05% vs. 1.84 ± 0.03%, *P* < 0.001). The *in vivo* efficacy of PL in reducing inflammatory cell infiltration in corneal tissue was further evaluated by immunohistochemistry. A high density of infiltrated neutrophils, macrophages, and CD4^+^ cells within the stromal layer of allografts were observed in vehicle-treated mice. However, PL treatment dramatically inhibited the local infiltration of these inflammatory cells mentioned above, as presented in **Figure 3C**. In parallel, the mRNA levels of major inflammatory factors, including IL-2 (*P* < 0.001), IFN-γ (*P* < 0.05), IL-17A (*P* < 0.05), TNF-α (*P* < 0.01) and IL-1β (*P* < 0.05) in PL-treated mice were significantly downregulated compared with vehicle-treated mice (**Figure 3D**).

### 3.4 PL exhibited anti-angiogenic capacity in HUVECs under hypoxic conditions

**Figure 4:**
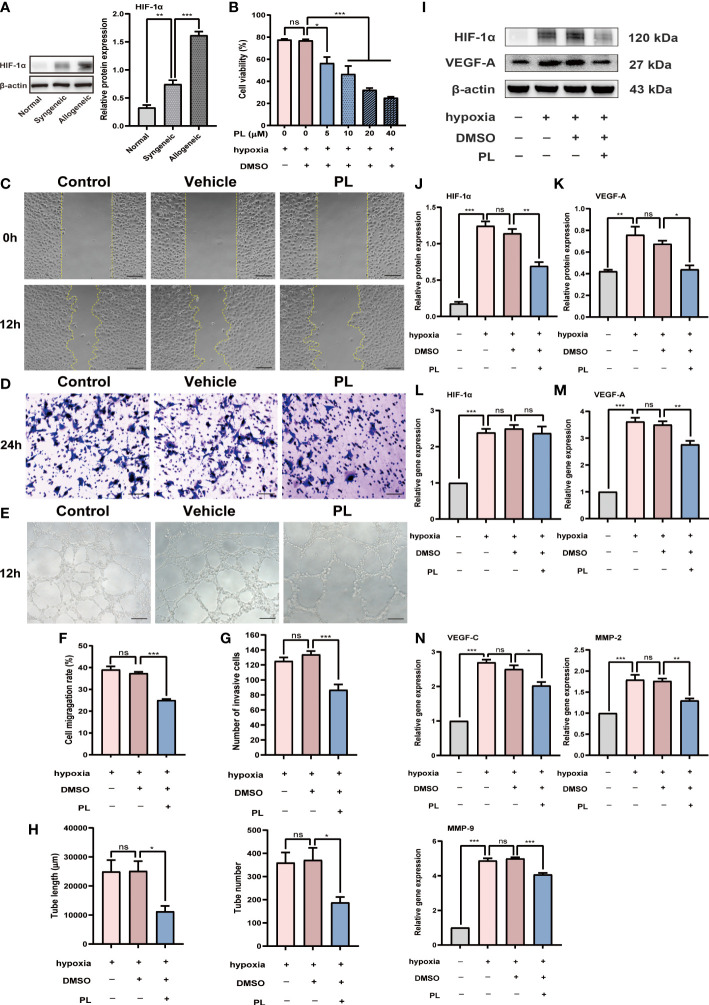
PL inhibited hypoxia-induced angiogenesis in HUVECs. **(A)** Representative Western blot images and semiquantitative analysis of the level of HIF-1α in the corneal grafts from normal, syngeneic, and allogeneic groups (n = 6/group). **(B)** HUVECs were incubated in the hypoxic incubator with nothing, vehicle (0.1% DMSO) or PL (5 μM, 10 μM, 20 μM or 40 μM). The effect of different concentrations of PL on HUVECs proliferation was assessed by a CCK8 assay. **(C)** Representative images of wound healing assay were taken at 0 h after scratch-wounding and 12 hours after PL treatment (5 μM). scale bar 100 µm. **(D)** Representative images of invasion assay were taken at 24 hours after PL treatment (5 μM). scale bar 50 µm. **(E)** Representative images of tube formation assay were taken at 12 hours after PL treatment (5 μM). scale bar 100 µm. **(F-H)** Quantitative analysis of wound healing, transwell invasion and tube formation assay in different groups. The tube length and tube number were assessed in tube formation assay. **(I-M)** HUVECs were incubated under normoxia or hypoxia with or without PL (5 μM). Hif-1α and VEGF-A protein and mRNA expression were measured by western blot and qRT-PCR. **(N)** The mRNA expression levels of VEGF-C, MMP-2 and MMP-9 were analyzed by qRT-PCR. Ns *P* > 0.05, *P < 0.05, **P < 0.01, ***P < 0.001.

One key pathological feature of cornea allograft rejection is angiogenesis, prominently triggered by hypoxia in other ocular surface disorders. To validate whether there is a hypoxic condition in corneal allograft rejection, we examined the expression of Hif-1α, a generally accepted evaluation indicator for hypoxia. Our result revealed that the protein expression level of HIF-1α was substantially raised in allogeneic mice when compared to syngeneic mice (**Figure 4A**, P < 0.001). To mimic the hypoxic environment during corneal allograft rejection, a cellular hypoxia model was established in HUVECs and was then used to investigate the role of PL on angiogenesis under hypoxic conditions *in vitro*. At first, a CCK-8 assay was performed to identify the optimal concentration for PL treatment *in vitro*. It was found that under hypoxic conditions, 5 μM of PL treatment could significantly inhibit the proliferation of HUVECs by 21%, and such inhibitory effect enlarged as the concentration of PL increased (**Figure 4B**). Thus, 5 μM of PL treatment was chosen during the following experiments unless otherwise specified. The migration and invasion of HUVECs in response to hypoxia were evaluated by the wound healing and transwell invasion assay. These results revealed that both the migration rate and invasion capacity of HUVECs treated with PL were significantly reduced compared with HUVECs treated with vehicle (**Figures 4C, D, F, G**, *P* < 0.001). A pronounced inhibition of the tube formation by PL treatment was noted (**Figure 4E**), as indicated by a decrease in the tube length and tube number (**Figure 4H**, both P < 0.05).

Intriguingly, we found that the expression of HIF-1α, a critical pro-angiogenic molecule in response to hypoxic stimulation, also clearly differed between PL-treated and vehicle-treated cells in protein level (**Figures 4I, J**, *P* < 0.01). This may provide one potential mechanism to explain the anti-angiogenic effect of PL under hypoxia. Nevertheless, it should be noted that the level of HIF-1α gene did not change following PL treatment (**Figure 4L**). As a major contributor to angiogenesis, VEGF-A expression was decreased to a similar extent by PL treatment both in mRNA (**Figure 4M**, *P* < 0.01) and protein (**Figure 4K**, *P* < 0.05) levels. Other angiogenic factors such as VEGF-C (**Figure 4N**, *P* < 0.05), MMP-2 (**Figure 4N**, *P* < 0.01), and MMP-9 (**Figure 4N**, *P* < 0.001) were also downregulated in mRNA level.

### 3.5 PL reduced expression of adhesion molecules and pro-inflammatory chemokines in HUVECs upon TNF-α stimulation

**Figure 5:**
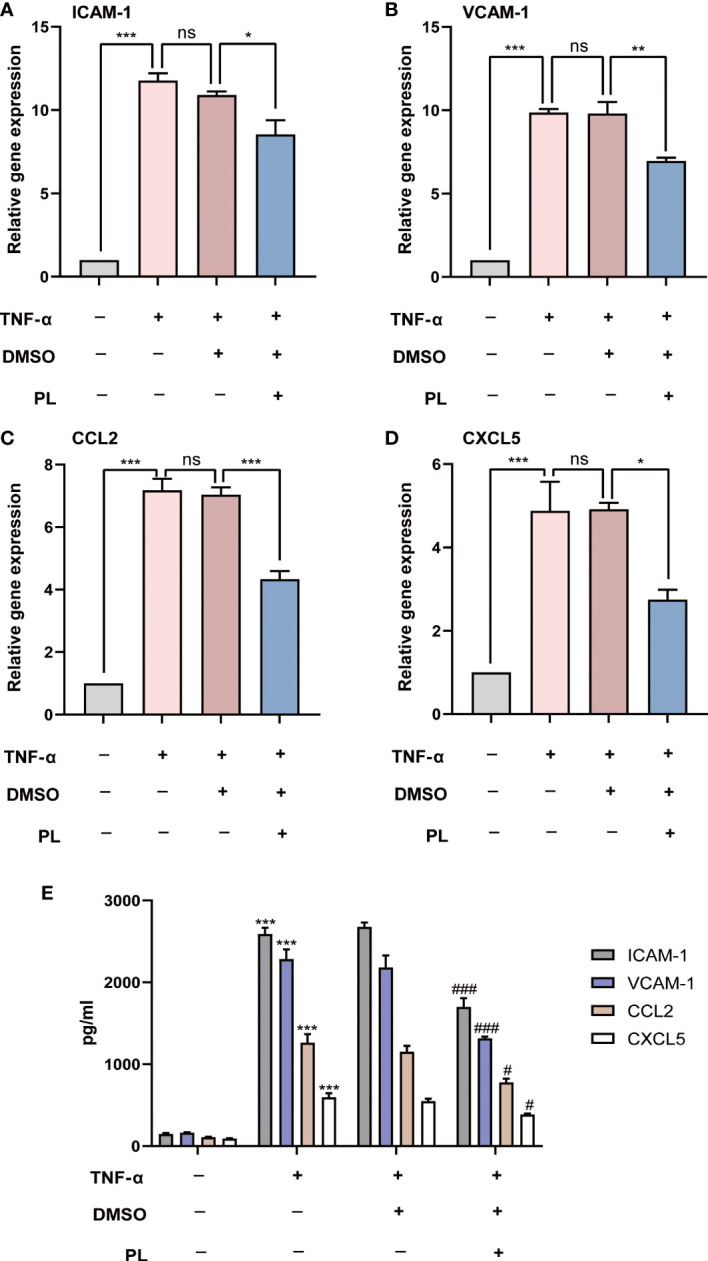
PL inhibited TNF-α-induced expression of adhesion molecules and pro-inflammatory chemokines in HUVECs. **(A–D)** HUVECs were incubated under normal condition or TNF-α stimulation with or without PL. The mRNA expression levels of ICAM-1, VCAM-1, MMP-2 and MMP-9 were analyzed by qRT-PCR. Ns *P* > 0.05, *P < 0.05, **P < 0.01, ***P < 0.001. **(E)** The secretions of ICAM-1, VCAM-1, MMP-2 and MMP-9 in different groups were detected by ELISA. #, *P < 0.05, ###, ***P < 0.001. (# compares TNF-α 20 ng/ml conditions with normal condition and * compares PL 5 μM condition with 0.1% DMSO condition).

In the next setting, we investigated the influence of PL on inflammation, another essential feature of corneal allograft rejection. HUVECs were stimulated with recombinant human TNF-α to induce the vascular inflammatory response. The adhesion molecules including intercellular adhesion molecule-1 (ICAM-1) and vascular cell adhesion molecule-1 (VCAM-1), as well as pro-inflammatory chemokines including chemokine (C–C motif) ligand 2 (CCL2; MCP-1) and C-X-C motif chemokine 5 (CXCL5; ENA78), which can facilitate leukocyte migration and recruitment, were measured using quantitative real-time PCR. We found that the mRNA level of ICAM-1, VCAM-1, CCL2, and CXCL5 substantially increased upon TNF-α stimulation. However, such elevation was significantly suppressed by PL treatment (**Figures 5A–D**). Consistently, the secretion levels of ICAM-1, VCAM-1, CCL2, and CXCL5 protein were also hindered by PL treatment (**Figure 5E**).

## 4 Discussion

The maintenance of corneal angiogenic privilege and suppression of ocular surface inflammation are required for successful corneal transplantation. Although PL has been demonstrated to have anti-angiogenic and anti-inflammatory potentials for tumor and autoimmune disease therapy (16, 17), no studies have been carried out on its role in transplant rejection, especially in corneal allograft rejection. The present study demonstrated, for the first time, the potential of PL as a novel and potent inhibitor of pathological neovascularization and inflammation in corneal allograft rejection.

In alloimmune response, neovascularization including angiogenesis and lymphangiogenesis, can play pivotal roles in inducing corneal allograft rejection (3). The newly formed blood and lymphatic vessels facilitate the transportation of immune cells and inflammatory mediators, which mediate the process of rejection (6). The inhibitory effect of PL on neovascularization has been verified using HUVECs culture under normoxia *in vitro* and the zebrafish embryo model *in vivo* (11). In the present study, we further found that PL can regulate HUVECs angiogenesis process in a hypoxic environment and attenuate corneal neovascularization in the murine corneal allograft transplantation model. One of the critical regulators of ocular neovascularization is HIF-1α, a well-established oxygen sensor, which is induced under hypoxic conditions and degraded under normoxic conditions (18). HIF-1α can regulate angiogenesis by several mechanisms; one such mechanism is to induce transcriptional activation of downstream pro-angiogenic molecules, especially VEGF (19, 20). In this context, we showed the specific elevation of HIF-1α expression in corneal allografts of the murine model, thereby providing the first demonstration of the hypoxic condition during corneal allograft rejection. Also, our results indicated that HUVECs in hypoxia could enhance HIF-1α expression, with a concomitant upregulated VEGF-A expression. This increase in HIF-1α/VEGF-A signaling pathway was reversed by PL treatment, implying that HIF-1α/VEGF-A signaling pathway may be one potential mechanism underlying the inhibitory effect of PL on corneal neovascularization. Of note, hypoxia-induced HIF-1α protein expression was inhibited by PL, while no apparent change in mRNA expression was detected. This finding suggested that PL may modulate HIF-1α protein expression in a post-transcriptional way, although it required further investigation to understand the in-depth mechanism. Additionally, we observed that PL inhibited the mRNA level of several other crucial pro-angiogenic factors, including VEGF-C, MMP-2, and MMP-9, further supporting the hypothesis that PL can regulate angiogenesis under hypoxia through HIF-1α-dependent transcriptional activation of downstream pro-angiogenic factors and eventually mitigate allograft rejection.

The inflammatory response is an essential step in allograft rejection (21). This pathological process involves the generation, activation, and recruitment of immune cells, releasing a variety of inflammatory mediators that contribute to tissue damage (22, 23). Attenuated inflammatory response after PL treatment has previously been reported. PL pretreatment mitigated inflammatory cell infiltration in the airway and reduced Th2-type cytokine expression in ovalbumin-induced asthma and airway inflammation (12). There was also evidence that PL exhibited anti-inflammatory effects *via* inhibiting macrophage activation in psoriasis-like skin inflammation (24). This study demonstrated that as major effectors of the inflammatory response in corneal allograft rejection (25), CD4^+^ T cells and macrophages in regional lymphoid tissues were inhibited by PL treatment, suggesting that PL attenuated the inflammatory response by inhibiting the generation of effector T cells and reducing APCs emigration to DLNs for antigen presentation. Additionally, a remarkable reduction in infiltration and inflammatory cytokine secretion of neutrophils, macrophages, and CD4^+^ T cells, can also support the anti-inflammatory capacity of PL in murine corneal allograft transplantation model. CCL2 is the crucial chemokine that drives the recruitment of monocytes/macrophages and T lymphocytes, and CXCL5 is a well-known neutrophil chemoattractant. Both of them can be secreted by vascular endothelial cells (26). Similarly, ICAM-1 and VCAM-1, the adhesion molecules involved in the migration of inflammatory cells, can also be expressed in vascular endothelial cells (27). Here, our results revealed that PL suppressed the production of the aforementioned chemokines and adhesion molecules in TNFα-stimulated HUVECs. This hinted that PL might affect inflammation by inhibiting the induction of pro-inflammation chemokines, and adhesion molecules in vascular endothelial cells. However, it should also be further investigated whether PL has direct impacts on diverse immune cell types and those inflammatory signaling cascades involved.

Summarily, in the hypoxic and inflammatory microenvironment of the ocular surface in allograft rejection, PL can exhibit anti-angiogenic and anti-inflammatory capacity, thereby prolonging corneal allograft survival. PL regulated angiogenesis under hypoxic conditions and HIF-1α/VEGF-A signaling pathway might be one potential mechanism underlying its inhibitory effect. Furthermore, PL suppressed the migration and infiltration of inflammatory cells to the allografts. Meanwhile, it also inhibited the pro-inflammation chemokine and adhesion molecular secretion of inflammatory neovascularization contributing to an inflammatory response. This study shows that PL offers the potential for alleviating rejection and prolonging corneal allograft survival.

## Data availability statement

The original contributions presented in the study are included in the article/supplementary material. Further inquiries can be directed to the corresponding authors.

## Ethics statement

The animal study was reviewed and approved by Animal Care and Use Committee of Eye & ENT Hospital (Shanghai, China).

## Author contributions

XF and TY: conceptualization and design. XF, JQ, and TY: investigation. XF: writing original draft. JZ: revision of the manuscript. JX: supervision and funding acquisition. All authors contributed to the article and approved the submitted version.

## References

[1] Hori J Yamaguchi T Keino H Hamrah P Maruyama K. Immune privilege in corneal transplantation. Prog Retin Eye Res (2019) 72:100758. doi: 10.1016/j.preteyeres.2019.04.002 31014973

[2] Mukwaya A Lindvall JM Xeroudaki M Peebo B Ali Z Lennikov A. A microarray whole-genome gene expression dataset in a rat model of inflammatory corneal angiogenesis. Sci Data (2016) 3:160103. doi: 10.1038/sdata.2016.103 27874850 PMC5119432

[3] Zhong W Montana M Santosa SM Isjwara ID Huang YH Han KY. Angiogenesis and lymphangiogenesis in corneal transplantation-a review. Surv Ophthalmol (2018) 63(4):453–79. doi: 10.1016/j.survophthal.2017.12.008 PMC6003844 29287709

[4] Li X Zhou Q Hanus J Anderson C Zhang H Dellinger M. Inhibition of multiple pathogenic pathways by histone deacetylase inhibitor SAHA in a corneal alkali-burn injury model. Mol Pharm (2013) 10(1):307–18. doi: 10.1021/mp300445a PMC3697033 23186311

[5] Ellenberg D Azar DT Hallak JA Tobaigy F Han KY Jain S. Novel aspects of corneal angiogenic and lymphangiogenic privilege. Prog Retin Eye Res (2010) 29(3):208–48. doi: 10.1016/j.preteyeres.2010.01.002 PMC3685179 20100589

[6] Dietrich T Bock F Yuen D Hos D Bachmann BO Zahn G. Cutting edge: lymphatic vessels, not blood vessels, primarily mediate immune rejections after transplantation. J Immunol (2010) 184(2):535–9. doi: 10.4049/jimmunol.0903180 PMC4725297 20018627

[7] Costa C Incio J Soares R. Angiogenesis and chronic inflammation: cause or consequence. Angiogenesis (2007) 10(3):149–66. doi: 10.1007/s10456-007-9074-0 17457680

[8] Li Z Chen J Lei L Jiang N Zhu Y Jia Y. Laquinimod inhibits inflammation-induced angiogenesis in the cornea. Front Med (Lausanne) (2020) 7:598056. doi: 10.3389/fmed.2020.598056 33244468 PMC7683777

[9] Leedom AJ Sullivan AB Dong B Lau D Gronert K. Endogenous LXA4 circuits are determinants of pathological angiogenesis in response to chronic injury. Am J Pathol (2010) 176(1):74–84. doi: 10.2353/ajpath.2010.090678 20008149 PMC2797871

[10] Sun LD Wang F Dai F Wang YH Lin D Zhou B. Development and mechanism investigation of a new piperlongumine derivative as a potent anti-inflammatory agent. Biochem Pharmacol (2015) 95(3):156–69. doi: 10.1016/j.bcp.2015.03.014 25850000

[11] Liu Y Chang Y Yang C Sang Z Yang T Ang W. Biodegradable nanoassemblies of piperlongumine display enhanced anti-angiogenesis and anti-tumor activities. Nanoscale (2014) 6(8):4325–37. doi: 10.1039/c3nr06599e 24622772

[12] Lu C Zhang B Xu T Zhang W Bai B Xiao Z. Piperlongumine reduces ovalbumin−induced asthma and airway inflammation by regulating nuclear factor−κB activation. Int J Mol Med (2019) 44(5):1855–65. doi: 10.3892/ijmm.2019.4322 PMC6777695 31485644

[13] Xiao Y Shi M Qiu Q Huang M Zeng S Zou Y. Piperlongumine suppresses dendritic cell maturation by reducing production of reactive oxygen species and has therapeutic potential for rheumatoid arthritis. J Immunol (2016) 196(12):4925–34. doi: 10.4049/jimmunol.1501281 27183580

[14] Fan X Zhang J Dai Y Shan K Xu J. Blockage of P2X7R suppresses Th1/Th17-mediated immune responses and corneal allograft rejection *via* inhibiting NLRP3 inflammasome activation. Exp Eye Res (2021) 212:108792. doi: 10.1016/j.exer.2021.108792 34656546

[15] Inomata T Mashaghi A Di Zazzo A Lee SM Chiang H Dana R. Kinetics of angiogenic responses in corneal transplantation. Cornea (2017) 36(4):491–6. doi: 10.1097/ICO.0000000000001127 PMC5334361 28060028

[16] Tripathi SK Biswal BK. Piperlongumine, a potent anticancer phytotherapeutic: Perspectives on contemporary status and future possibilities as an anticancer agent. Pharmacol Res (2020) 156:104772. doi: 10.1016/j.phrs.2020.104772 32283222

[17] Ranjan A Ramachandran S Gupta N Kaushik I Wright S Srivastava S. Role of phytochemicals in cancer prevention. Int J Mol Sci (2019) 20(20):4981. doi: 10.3390/ijms20204981 31600949 PMC6834187

[18] Fu ZJ Wang ZY Xu L Chen XH Li XX Liao WT. HIF-1α-BNIP3-mediated mitophagy in tubular cells protects against renal ischemia/reperfusion injury. Redox Biol (2020) 36:101671. doi: 10.1016/j.redox.2020.101671 32829253 PMC7452120

[19] Wong BW Marsch E Treps L Baes M Carmeliet P. Endothelial cell metabolism in health and disease: impact of hypoxia. EMBO J (2017) 36(15):2187–203. doi: 10.15252/embj.201696150 PMC5538796 28637793

[20] Tirpe AA Gulei D Ciortea SM Crivii C Berindan-Neagoe I. Hypoxia: Overview on hypoxia-mediated mechanisms with a focus on the role of HIF genes. Int J Mol Sci (2019) 20(24):6140. doi: 10.3390/ijms20246140 31817513 PMC6941045

[21] Rao Z Sun J Pan X Chen Z Sun H Zhang P. Hyperglycemia aggravates hepatic ischemia and reperfusion injury by inhibiting liver-resident macrophage M2 polarization *via* C/EBP homologous protein-mediated endoplasmic reticulum stress. Front Immunol (2017) 8:1299. doi: 10.3389/fimmu.2017.01299 29081777 PMC5645540

[22] Ochando J Ordikhani F Boros P Jordan S. The innate immune response to allotransplants: mechanisms and therapeutic potentials. Cell Mol Immunol (2019) 16(4):350–6. doi: 10.1038/s41423-019-0216-2 PMC6462017 30804476

[23] Taylor AW. Ocular immune privilege and transplantation. Front Immunol (2016) 7:37. doi: 10.3389/fimmu.2016.00037 26904026 PMC4744940

[24] Thatikonda S Pooladanda V Sigalapalli DK Godugu C. Piperlongumine regulates epigenetic modulation and alleviates psoriasis-like skin inflammation *via* inhibition of hyperproliferation and inflammation. Cell Death Dis (2020) 11(1):21. doi: 10.1038/s41419-019-2212-y 31924750 PMC6954241

[25] Amouzegar A Chauhan SK Dana R. Alloimmunity and tolerance in corneal transplantation. J Immunol (2016) 196(10):3983–91. doi: 10.4049/jimmunol.1600251 PMC4874505 27183635

[26] Iturriaga-Goyon E Buentello-Volante B Magaña-Guerrero FS Garfias Y. Future perspectives of therapeutic, diagnostic and prognostic aptamers in eye pathological angiogenesis. Cells (2021) 10(6):1455. doi: 10.3390/cells10061455 34200613 PMC8227682

[27] Jian D Wang Y Jian L Tang H Rao L Chen K. METTL14 aggravates endothelial inflammation and atherosclerosis by increasing FOXO1 N6-methyladeosine modifications. Theranostics (2020) 10(20):8939–56. doi: 10.7150/thno.45178 PMC7415798 32802173

